# Global Challenges: Global Health

**DOI:** 10.1002/gch2.1006

**Published:** 2015-09-21

**Authors:** Steven J. Hoffman, John‐Arne Røttingen

**Affiliations:** ^1^ Global Strategy Lab Faculty of Law, University of Ottawa Ottawa Ontario Canada; ^2^ Division of Infectious Disease Control Norwegian Institute of Public Health Oslo Norway

## Abstract

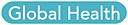

Global health is a relatively new field and area of practice. For centuries, people have worried about disease and medical misfortune coming from afar, inspiring numerous international health and tropical medicine initiatives. But with hyper‐globalization of the last few decades, we have begun to think of health as truly global. Health is increasingly recognized as something requiring collaboration and solidarity across traditional boundaries.

Indeed, crisis after crisis has shown that we live in an interdependent world where health and well‐being are shaped by circumstances, decisions, and events occurring in distant places: pandemics spread between countries within days; health professionals emigrate from countries where they trained and are most needed; drivers of unhealthy lifestyles shift from one place to another; food supply lines are globally integrated; and environmental health risks like climate change defy national borders. Today, the risks and potential rewards of globalization for health are great, but managing these risks and reaping these rewards depend on the effective management of globalization by the world's governments, civil society organizations, businesses, and international institutions.

The good news is that global population health is improving. The past 25 years have witnessed a particularly golden era of unprecedented health achievements. Child mortality has been cut by 47% and maternal mortality by 45%; and the spread of HIV/AIDS, malaria, and other diseases is starting to reverse (Frenk & Hoffman, 2015). While much of this progress is likely attributed to external factors and circumstances, explicit global health action has certainly made a difference. It is what has allowed access to insecticide‐treated bed nets, tougher government regulations on tobacco, and new vaccines for diseases like meningitis, rotavirus, and Ebola. Global investments in development assistance for health have skyrocketed from $US6.86 billion in 1990 to $US35.9 billion in 2014 (IHME, 2015). The results are remarkable.

But at the same time, these impressive health gains have not been shared equally, and we still have far to go. Extreme poverty and inequality remain at unacceptably high levels in richer and poorer societies alike, thereby representing major threats to global health as well as direct consequences of ill health. Data unsurprisingly show that ill–health is unfairly concentrated in low and middle‐income countries. According to United Nations figures, 768 million people around the world drink from unsafe water sources, 870 million suffer chronic undernourishment, and 2.5 billion lack basic sanitation. Six million children die each year from avoidable causes, 52 million mothers annually experience labor without skilled attendants, and 222 million women lack access to effective contraception. The fact that 1.2 billion people currently live in extreme poverty threatens peace, prosperity, and human potential (Frenk & Hoffman, 2015). Health and well‐being indicators represent some of the best concrete measures of development. One must only consider how a person born today in Cameroon can expect to live 55 years, while a neonate in Canada can expect 81 years of life (World Bank, 2015). Such tragic inequities justify continuing the global health community's special focus on improving health in the least well‐off places and among the worst‐off population groups, whether that is impoverished Haiti, populous China, or the powerless poor living in the most prosperous places.

Successfully addressing these global health challenges requires more than just new technology, or more medical care, or better trained health workers. It requires new knowledge and social innovations for how we govern our global society, how we finance health interventions, and how we deliver on the promise of what we already know. Global health has its roots in medicine and public health; while these disciplines will surely remain the bedrock of global health for years to come, the field has now matured to the point where it needs new perspectives that cross disciplinary divides in ways that can be maximally helpful for solving the tough global health problems we face. Such interdisciplinary perspectives on global health challenges that cross anthropology, biology, economics, engineering, epidemiology, geography, law, management, philosophy, politics, sociology, statistics and virology – and many more! – need a home where they will be welcomed, celebrated, criticized, and conveyed in whatever way will amplify their potential impact.

*Global Challenges* is that new home. As founding chief editors, we are thrilled to help build this new peer‐reviewed journal into a premier publication platform by curating important conversations that matter for the future of global health.

Three facts convinced us to lead this initiative. The first was the absence of high‐quality platforms for truly interdisciplinary scholarship, analysis, and commentary on the explicitly global dimensions of health challenges. There are many existing journals that publish global health research, but most are rooted in a single discipline and require manuscripts to conform to the norms of that discipline. This means that too often, the content and form of published knowledge is shaped by what journals will allow to be disseminated rather than what is best for advancing knowledge and what may be most helpful for practice and policy. We see this as an opportunity for creating a truly interdisciplinary platform, one that embraces policy‐relevant research and commentary and respects the unique and widely differing disciplines from which authors come.

The second was the many untapped opportunities for cross‐sectoral learning that a mega‐journal like *Global Challenges* could offer. In undertaking this initiative, we are essentially bringing together five new journals focused on five different global challenges under a common branding and editorial banner. This means that articles published in the journal will have audiences across sectors and that global health scholars, practitioners, and policymakers following the journal will get a glimpse of the exciting conversations taking place in other areas that are being curated by other chief editors. So much of global health depends on our environment, energy, food/agriculture, and water. Likewise, there is so much that we can learn from scholars, practitioners, and commentators working in these other sectors. Too often, we are divided by the work we do in siloes; *Global Challenges* represents one additional effort to break down these artificial sectoral barriers.

The third fact was our publisher's commitment to making *Global Challenges* both open access – freely available to all – and one of its flagship journals. Wiley is investing considerable resources into this venture. Such investments will yield real dividends for the authors who publish with us, the reviewers who help us allocate limited journal space, and the practitioners and policymakers who we hope will eventually count on the journal for learning about bold new ideas with potentially big impact.

From the beginning, we have been working with Wiley to ensure this journal takes knowledge translation seriously. This means that the journal will be taking additional proactive measures beyond the ordinary to ensure that research findings have the greatest chance of reaching those decision‐makers who can act upon them. For example, we are very excited to craft special series on complex policy challenges, especially in rapid response to real‐world policy events that are quickly unfolding. Authors can expect faster publishing timelines, and readers will see research, analysis, and commentary at times when it is needed and most relevant.

To make all of this happen, we are thrilled to be working with a great team of editors who are actively in the throes of their own ambitious research programs and at the cutting edge of translating research findings into policy impact. Our senior editorial team includes health economist Karen Grépin (New York University), public health lawyer Benn McGrady (World Health Organization), global health governance scholar Suerie Moon (Harvard University), physician and medical ethicist Ole Frithjof Norheim (University of Bergen), political scientist Lauren Paremoer (University of Cape Town), and physician and health policy analyst Gavin Yamey (Duke University). We expect this small team to expand – geographically, thematically, and disciplinarily – both in the short term and as future needs arise.

Ultimately, the success of this effort will depend on you: our authors, reviewers, readers, and friends. *Global Challenges* aims to be recognized as a leading source of empirical studies, evidence syntheses, critical analyses, and important commentaries that address transnational health threats and social inequalities requiring global collective action. We are counting on you to make this endeavor a success – which we firmly believe will benefit the field of global health and the complex world in which we live. For authors, we welcome manuscripts on all global health challenges, especially those that use interdisciplinary, mixed method, and/or new approaches. We look forward to working with you in the years to come.

## References

[gch21006-bib-0001] Frenk J, Hoffman S.J. (Eds.) “To Save Humanity”: What Matters Most for a Healthy Future. Oxford University Press: New York, 2015; pp. xix–xxi.

[gch21006-bib-0002] IHME . Financing Global Health 2014: Shifts in Funding as the MDG Era Closes. Institute for Health Metrics & Evaluation: Seattle, WA, 2015 Available at: http://www.healthdata.org/policy‐report/financing‐global‐health‐2014‐shifts‐funding‐mdg‐era‐closes.

[gch21006-bib-0003] World Bank . World Development Indicators 2015. World Bank: Washington DC, 2015 Available at: http://data.worldbank.org/products/wdi.

